# Supplementary material for: Exogenous Arachidonic Acid Affects Fucoxanthin Biosynthesis and Photoprotection in Phaeodactylum tricornutum
Source: Mar Drugs. 2022 Oct 17;20(10):644. doi: 10.3390/md20100644 (PMC9605588; doi:10.3390/md20100644)
Supplement: Supplementary file 1 [file marinedrugs-20-00644-s001.zip › marinedrugs-1914805-supplementary.pdf]

**Supplementary Information for**

# Exogenous Arachidonic Acid Affects Fucoxanthin Biosynthesis and Photoprotection in *Phaeodactylum tricornutum*

Shuaiqi Zhu [1, 2], Song Bin[2], Shan Lu[1*], Wenqiang Yang [2, 3, 4*]

**Affiliations:**

[1]State Key Laboratory of Pharmaceutical Biotechnology, School of Life Sciences, Nanjing University, Nanjing, 210023, China

[2]Photosynthesis Research Center, Key Laboratory of Photobiology, Institute of Botany, Chinese Academy of Sciences, Beijing, 100093, China

[3]University of Chinese Academy of Sciences, Beijing, 100049, China

[4]Innovative Academy of Seed Design, Chinese Academy of Sciences, Beijing 100093, China

[*]Correspondence: shanlu@nju.edu.cn (S.L.); wqyang@ibcas.ac.cn (W.Y.);

Tel.: +86-18651613471(S.L.); +86-18211169660 (W.Y.)

**Supplemental Table S1.** Primers used for RT-qPCR in this study.

| Gene name | Accession number | Primer sequence (5'-3') |
|---|---|---|
| *β-ACTIN* | AY_713398.1 | ACT-F: AGACCATTATGAAGTGCGAT<br>ACT-R: ACCCTCCAATCCAAACAG |
| *LCYB* | XM_002176576.1 | LCYB-F: CCCCAATAACGACAAACACG<br>LCYB-R: CAAAGAACGGCGTCACAGAT |
| *PSY* | XM_002178740.1 | PSY-F: ATCCCACCTTGGACATTACG<br>PSY-R: GTTCACTAGCATCTTCGCCTA |
| *PDS* | XM_002184476.1 | PDS-F: TTGCTCCGAATACGAATGTG<br>PDS-R: CACCACCCACGACGATAACT |
| *CRTISO* | XM_002179795.1 | CISO-F: AGATGATGTGACAGCCGAGC<br>CISO-R: TTTTCGTCCTTTAGTTCGTTGTAG |
| *ZDS* | XM_002176649.1 | ZDS-F: GATTGCCTACGCTTTGGG<br>ZDS-R: GCATGGGCTGCTAAGATTGA |
| *ZEP* | XP_002180238 | ZEP-F: GCAATGGTTCGCACTGATAC<br>ZEP-R: GTAGGCAAAGTCATCCCACA |
| *LHCX1* | XP_002179760.1 | LHCX1-F: CCTTGCTCTTATCGGCTCTG |

| | | |
|---|---|---|
| | | LHCX1-R: ACGGTATCGCTTCAAAGTGG |
| *LHCX2* | XP_002176987.1 | LHCX2-F: CAGCACTAATGCCGCTTTCG<br>LHCX2-R: CGTGAGTAACTTCCGCTTCC |
| *LHCX3* | XP_002178699.1 | LHCX3-F: TCCCGTTGGTATCTTTGATCC<br>LHCX3-R: GAAGATCCTTCCACGGCTTC |
| *LHCX4* | XP_002182760.1 | LHCX4-F: TCTTTGATCCACTCCGCTTC<br>LHCX4-R: GGCGTTCCATAGAAAGTTCG |
